# Motivations and Limitations Associated with Vaping among People with Mental Illness: A Qualitative Analysis of Reddit Discussions

**DOI:** 10.3390/ijerph14010007

**Published:** 2016-12-22

**Authors:** Ratika Sharma, Britta Wigginton, Carla Meurk, Pauline Ford, Coral E. Gartner

**Affiliations:** 1 School of Public Health, The University of Queensland, Herston, QLD 4006, Australia; b.wigginton@uq.edu.au (B.W.); c.meurk@uq.edu.au (C.M.); c.gartner@uq.edu.au (C.E.G.); 2 Policy and Epidemiology Group, Queensland Centre for Mental Health Research, Locked Bag 500, Archerfield, QLD 4018, Australia; 3 School of Dentistry, The University of Queensland, Herston, QLD 4006, Australia; p.ford1@uq.edu.au

**Keywords:** social media, mental disorders, smoking cessation, harm reduction, qualitative research, electronic cigarettes, Reddit, vaping

## Abstract

This study aims to understand the nature and significance of online lay discussions about e-cigarettes and mental illness. We systematically searched the website Reddit.com using keywords related to e-cigarettes and mental illness. We coded relevant posts into themes under the framework of motivations for and limitations of vaping for people with mental illness. The thematic analysis included 3263 comments from 133 discussion threads. Six themes were classified as motivations to vape for people with mental illness: Self-medication; Quitting smoking; Freedom and control; Hobby; Social connectedness; and Motivation from caregivers and online communities. The limitations of vaping included: Unsatisfactory substitute for cigarettes and psychiatric medicines; Drug interactions; Nicotine addiction; Risks of e-liquid; Practical difficulties and Cost. People with mental illness; and their carers; use online discussion boards like Reddit to discuss the benefits and limitations of e-cigarettes for people with mental illness. Both positive and negative views exist. Media platforms like Reddit may shape the opinions of stakeholders and generate lay expertise about contentious health topics such as e-cigarettes. These findings have implications for policy and practice concerning assisting smokers with mental illness to reduce their health risk through switching to e-cigarettes.

## 1. Introduction

Smoking prevalence among people with mental illnesses such as schizophrenia, bipolar disorder, depression and anxiety disorder, is much higher than among those without mental illness [1,2,3]. Higher rates of smoking combined with lower access to health care and socio-economic disadvantage contributes to the 10–15-year lower life expectancy of people with severe mental illnesses compared to the general population [4,5,6]. Encouraging smoking cessation is central to improving the physical health and life expectancy of people with mental illness [7]. However, smoking cessation rates among people with mental illness remain low and only limited evidence supporting the effectiveness of traditional smoking cessation treatments such as nicotine replacement therapy, varenicline and bupropion among people with severe mental illnesses exists [7,8,9].

There is growing interest in e-cigarettes, also known as nicotine vaporizers, as a tobacco harm reduction approach for those who are unable to quit smoking with conventional quit smoking methods [10]. E-cigarettes are battery operated devices that heat a solution of propylene glycol and/or vegetable glycerol, typically containing nicotine and flavorings to produce an aerosol which can be inhaled into the lungs [10]. A review of the evidence on e-cigarettes by the Royal College of Physicians concluded that while likely to be more harmful than nicotine replacement therapy, the risks of vaping are substantially lower than smoking [11]. Considering the difficulties people with mental illnesses experience in quitting smoking due to their higher nicotine dependence, inadequate smoking cessation support and greater exposure to pro-smoking environments, tobacco harm reduction approaches, such as switching from cigarettes to e-cigarettes, have been proposed to reduce smoking related harm in this priority population [12].

Use of e-cigarettes, known as vaping, is growing among people with mental illnesses [13]. A survey of 355 smokers with mental health conditions, conducted by Action on Smoking and Health (ASH) in the UK found that 28% of participants had used an e-cigarette to make a quit attempt [14]. Studies have shown that e-cigarettes are acceptable to smokers with mental illness and may assist them to quit or reduce smoking [15,16,17]. This research suggests that smokers with mental illness might benefit from e-cigarettes and substantial proportions are already using them. However, there is little research on how people with mental illness find information about e-cigarettes or on what type of information about e-cigarettes they seek. 

For emerging issues, such as e-cigarettes, advice from health practitioners may not be forthcoming. Surveys of health practitioners have found that many are unsure about the safety and efficacy of e-cigarettes for reducing smoking-related harms [18,19,20]. There is also a lack of clear and unequivocal official guidance concerning use of e-cigarettes for smoking cessation in most countries (the UK is a notable exception). In the absence of a consensus among medical professionals, people with mental illness may be compelled to rely on other information sources, including forms of “lay expertise” that are produced via participatory media platforms [21,22]. 

Online health information may be of special utility to smokers with mental illness, as they face the dual stigma of mental illness and smoking and also significant barriers to accessing mainstream health services [23,24,25,26]. Online social media may transcend these barriers by providing social support, reducing the isolation and facilitating adoption of health behaviors including smoking cessation and harm reduction behaviors [27,28]. Online discussion forums such as Reddit may provide a platform for peer-to-peer knowledge transfer about e-cigarettes for people with mental illness [29]. 

Reddit is the 29th most visited website in the world [30] and draws users from more than 200 different countries [31]. It is used as an online bulletin board and social media website [32]. Special forums called subreddits exist within the Reddit, to provide a virtual space dedicated to discussions about a particular topic [33]. Past research has investigated online forums and social media websites such as YouTube, Twitter and Reddit to analyze the information flow surrounding e-cigarettes in the general population [34,35,36,37]. However, to our knowledge no study has explored online social media dialogue around vaping by people with mental illness. Moreover, despite increasing use of e-cigarettes among people with mental illness, we do not know what factors act as motivators or barriers for uptake of vaping by this population group. This study aimed to examine the Reddit discussions about the use of e-cigarettes by people with mental illness with a focus on the motivations and limitations of e-cigarette use by this priority population group.

## 2. Materials and Methods

### 2.1. Sample and Data Collection

The content included in this analysis arose from a systematic search of Reddit online discussions, freely available in the public domain. The website (www.reddit.com) was searched by Ratika Sharma in April 2016 using the search terms “electronic cigarettes”, “e-cigarettes” and “vaping” in combination with the terms “psychiatric”, “mental illness”, “schizophrenia”, “depression”, “psychosis”, “anxiety” and “bipolar”. 

Posts were sorted using the “relevance” and “all time” functions available in the Reddit search engine to retrieve the most relevant posts from all points in time. All posts identified in the search were screened for relevance by reading the title and content of the initial discussion thread. Posts were included for analysis if the poster (someone who posts a comment on the Reddit website) of the initial thread discussed e-cigarette use in the context of mental illness, regardless of the diagnosis of mental illness (if any) of the poster. During the initial screening of the posts, we removed any posts which discussed vaping only in the context of cannabis by screening for words like marijuana, weed etc. Relevant primary posts and the ensuing discussions were imported into the qualitative data analysis software NVivo (Version 10, 2012, QSR International Pty Ltd., Melbourne, Australia) and Microsoft Excel for data coding, management and analysis. Metadata such as username, date of post, comment and subreddit name for each individual thread included in the analysis were also extracted into Microsoft Excel.

### 2.2. Analysis

We conducted a thematic data analysis, framed by our focus on the motivations for and limitations of vaping for people with mental illness [38]. Ratika Sharma read the posts many times to gain familiarity with the data and to identify initial patterns. Each poster was given a unique poster ID (PI). Open coding was performed to generate the maximum number of possible codes regarding reasons that motivated or discouraged posters from using e-cigarettes. The posts were coded into multiple categories where applicable. A random selection of discussion threads (10%, *n* = 13) was read and coded independently by Britta Wigginton in order to assess any potential discrepancies in coding (of which none were identified) and further develop codes. These codes were grouped into themes under the broad framework of motivators for and limitations of taking up vaping. The themes and associated codes were discussed among the research team to reach consensus and any disagreements were resolved through this discussion. This study received ethical clearance from the Human Research Ethics Committee of the School of Public Health, The University of Queensland (RS14022016).

## 3. Results

A total of 3263 comments from 133 discussion threads, 1681 individual poster IDs and 21 subreddits were analysed (see Table S1). As seen in Table 1 and Supplementary Table S2, around a quarter (24.6%, *n* = 413) of the posters self-identified as living with a mental illness, including schizophrenia (2.1%), depression (28.6%) and anxiety (34.9%); 21.8% reported having multiple diagnoses. Twelve posters (0.7%) identified as being mental health practitioners and 30 (1.8%) were a friend or relative of a person with mental illness. More than half of the posters identified as being vapers (52.3%) while 28.9% of the posters said that they were current (*n* = 41) or former smokers (*n* = 444).

Discussion threads were usually initiated with a post seeking information or with the purpose of sharing the poster’s experience with the wider Reddit community as a form of social dialogue or to help others. Posters often described their personal experiences of vaping and other quit smoking methods in the form of anecdotes, humour and internet acronyms. A small number of posts were from caregivers (family, friends and health practitioners) of people with mental illness. These posters were seeking information about e-cigarettes in order to introduce vaping to their family members, friends or patients with mental illness to help them improve their health by quitting smoking. Some posters talked about their family members who were able to quit smoking by switching to vaping. 

Six themes were classified as motivations to vape for people with mental illness: (1) Self-medication; (2) Quitting smoking; (3) Freedom and control; (4) Hobby; (5) Social connectedness; and (6) Encouragement from caregivers. The limitations or barriers to vaping included: (1) Unsatisfactory substitute for cigarettes and psychiatric medicines; (2) Drug interactions; (3) Nicotine addiction; (4) Risks of e-liquid; (5) Practical difficulties; and (6) Cost. Representative excerpts for each theme appear in Table 2 and Table 3 and Supplementary Tables S3 and S4.

### 3.1. Motivations for Using E-Cigarettes

#### 3.1.1. Self-Medication

Posters discussed the benefits of nicotine to alleviate psychiatric symptoms, similar to psychiatric drugs and/or cigarettes. Vaping was reported to provide relief from stress, anxiety, depression, intrusive thoughts and to improve mood. The users of e-cigarettes were usually ex-smokers who had switched from smoking to vaping. However, there were several cases of non-smokers taking up vaping in order to self-medicate their mental illness with nicotine. 

Many of those using nicotine for relief of mental illness symptoms expressed their dislike for prescription medications and believed that vaping was a better way to medicate their symptoms. One poster described prescription medication as affecting the “brain’s natural chemistry”. Vaping was put forth as a replacement for psychiatric drugs and an alternative to being “medicated all the time” with prescription drugs. Additionally, vaping was reportedly used to offset the adverse effects of psychiatric drugs such as lithium. 

#### 3.1.2. Quitting Smoking

E-cigarettes were described as a healthier alternative to a cigarette because of their ability to deliver nicotine without smoke. Cigarettes were often framed as a dangerous product associated with illness and death and referred to as “death sticks” and “cancer sticks”. On the other hand, vaping was most often described in innocuous terms, such as a “hobby”. Many posters acknowledged that existing research was inadequate to make claims about the absolute safety of e-cigarettes. However, they considered vaping to be a reduced harm option which was the “lesser of two evils” compared to tobacco cigarettes. Smoking reduction or switching completely to vaping was reported as resulting in a number of health benefits, including intertwined mental and physical benefits. Saving money was also mentioned as motivating a switch from smoking to vaping, which a number of posters described as being cheaper in the long run.

Some posters preferred vaping over traditional quit smoking methods such as nicotine replacement therapy and prescription stop smoking medications (e.g., varenicline and bupropion) because they had tried using these cessation aids without success, they had experienced adverse effects or they encountered reluctance to prescribe cessation medications from medical practitioners. Some posters described nicotine replacement therapy products and prescription cessation medications as aggravating their psychiatric symptoms. 

#### 3.1.3. Freedom and Control

Vaping was perceived as offering more control to vapers compared to smoking cigarettes and using psychiatric medications. A few posters who had taken up smoking in the past as a way of alleviating symptoms of mental illness and who now wanted to stop smoking, felt that vaping gave them more control by letting them choose the amount of nicotine they put in the vaporiser and helping them quit a dangerous habit (smoking). One poster also described vaping as a life saver that had provided them with freedom from their mental illness.

#### 3.1.4. Hobby

Hobbyist aspects of vaping, such as building coils (part of an e-cigarette which heats the e-liquid) and exploring different e-liquid flavours provided some posters with a distraction from their symptoms of mental illness. Vaping was described as a helpful diversion which helped them practice deep breathing, eat healthily and addressed their “oral fixation” or need for oral stimulation. Some vapers with mental illness reported improvement in their symptoms of mental illness, even though they reported using nicotine free e-liquids (0 mg nicotine), presumably due to the distraction vaping provided.

#### 3.1.5. Social Connectedness

Vaping was reported as providing mental health benefits by increasing online and offline social connectedness. Having a shared interest with other vapers that facilitated online interaction instilled a sense of being a part of a larger community. Social interactions also stemmed from curiosity about e-cigarettes among non-vapers. One poster described e-cigarettes as a “conversation starter” which reduced their social anxiety.

#### 3.1.6. Motivation by Caregivers and Online Communities

Posts suggested that people with mental illness are encouraged to take up vaping by caregivers, family members, health practitioners and online communities. For example, some posters stated that they started using e-cigarettes when they observed other vapers benefitting from them while others indicated that it was their caregivers who encouraged them to switch to e-cigarettes. These comments were corroborated by posts from health practitioners and caregivers that indicated their active support of their patients and family members with mental illness using e-cigarettes to quit smoking. Online communities such as vaping subreddits were also explicitly cited as an important source of information and encouragement for taking up vaping.

### 3.2. Limitations or Barriers to Using E-Cigarettes

#### 3.2.1. Unsatisfactory Substitute for Cigarettes and Psychiatric Medications

Comparisons were drawn between vaping and smoking cigarettes by posters who had experience with both. A small minority of posters were dissatisfied with the experience of vaping because e-cigarettes delivered only nicotine and not the other chemicals (“chemicals in burnt tobacco”) which a combustible cigarette provides. Combustible cigarettes were viewed as providing additional antidepressant effects due to the presence of certain other chemicals in tobacco smoke that are absent from e-cigarette vapour. A weaker nicotine “buzz” from e-cigarettes was attributed to the absence of these chemicals, thus making switching from smoking to vaping difficult. 

In response to those who expressed a preference for e-cigarettes over psychiatric medications, other posters disapproved of vaping as a replacement for psychiatric medication. They advised others that it is best to consult health professionals and use prescribed medications to alleviate their symptoms and not depend on e-cigarettes for self-medication. Some cautioned that vaping should only be treated as an adjunct to psychiatric treatment and not a replacement for it.

#### 3.2.2. Drug Interactions

Some posters were concerned about vaping potentially having adverse interactions with psychotropic medications. In some cases, this stemmed from health warnings and labels that accompanied e-liquids. One poster, who identified as being a pharmacist, advised that a synergistic stimulant effect between nicotine and antidepressant medications may lead to adverse effects such as suicidal ideation. However, vapers who used psychotropic medications reported that they had not experienced any adverse side effects whilst still encouraging others to consult their psychiatrist. 

#### 3.2.3. Nicotine Addiction

Although vaping was supported for its role in relieving anxiety and depression, some posters exhibited wariness of acquiring or maintaining nicotine addiction through vaping. Some posters believed that nicotine caused anxiety rather than cured it and that quitting all forms of nicotine would lead to better mental health. Non-smokers were mostly discouraged from taking up vaping nicotine and were advised to either use nicotine-free e-liquids and/or to seek medical help for their psychiatric symptoms. Several posters explicitly cautioned that vaping should only be treated as a “stopgap measure” until one stops smoking completely. Nicotine was also described by some as “nasty stuff” which causes cardiovascular disease and death. 

#### 3.2.4. Risks of E-Liquids

Toxicity by overdosing or intentionally drinking nicotine e-liquid was perceived as a potential threat to the safety of vapers with mental illness. Caregivers were especially concerned about e-cigarette use by those with self-harm tendencies. Physical adverse effects like burning mouth, sore throat and chest pains were also reported by some posters. Some posters admitted that although vaping potentially carries reduced health risks compared to smoking cigarettes, there is a paucity of research to enable conclusive statements to be made about its safety. Some posters stated that they were being persuaded by others to stop using e-cigarettes because of the unknown side effects of vaping. 

#### 3.2.5. Practical Difficulties

Posters discussed the potential problems and apprehensions associated with the use of e-cigarettes by people with mental illness, such as difficulty in learning how to use and maintain the vaping equipment. The posters who were interested in introducing vaping to their clients and family members wanted information about an efficient and easy to use e-cigarette. The most common perceived limitation of vaping was refilling the tank with e-liquid. Many posters wanted information about which devices were safest and easiest for a novice. Spilling nicotine e-liquid on skin while filling the vaporiser tank or accidentally swallowing the e-liquid were major concerns with respect to use of nicotine e-liquid by people with mental illness. These queries were addressed by posters who said that small amounts of nicotine e-liquid would not cause more than a “wicked buzz” or “tingly” sensation if swallowed or spilled on skin. One poster reported developing a “callous” on their skin after being in contact with nicotine e-liquid but no other adverse effect. Using pre-filled cartridges was advised for those who do not possess adequate ability to refill a tank and also to avoid spillage and swallowing of e-liquid.

#### 3.2.6. Cost

Cost of initial purchase of vaping equipment was another concern that was discussed frequently. While vaping was seen as more economical than purchasing cigarettes in the long run, some posters had misspent money on experimenting with different devices and e-liquids. One mental health worker expressed concern that trying out an e-cigarette was a costly exercise unlikely to be undertaken by their underprivileged clients. Finding easily accessible e-cigarette retailers was also identified as a practical difficulty for some persons with mental illness.

## 4. Discussion

The results give insight into the spontaneously generated views and perspectives of a sample of people with mental illness, their caregivers and others in the Reddit community, such as vapers. Our findings indicate the nuanced deliberations, both for and against people with mental illness taking up vaping. Some symmetry was evident in these deliberations insofar as themes that motivated some people to use e-cigarettes (e.g., cost, health, or as an alternative to psychotropic medications) were also framed by others as reasons to not use e-cigarettes. Reasons given for being interested in vaping, namely, as a smoking cessation aid, a form of self-medication and as a hobby reflect those found in other studies which show that e-cigarettes are primarily used for harm reduction, quitting smoking and sensory-behavioral benefits [39,40]. Sensory satisfaction and rituals associated with e-cigarettes are important determinants for their use. Boredom and conditioned cues for smoking are two reasons why people with mental illness smoke and smoking cessation methods that address these aspects may be particularly effective for this population [16,41,42,43].

E-cigarettes were viewed as empowering to users by providing them control over their nicotine dose, psychiatric medications, health and finances. Vaping was also seen as having social benefits which are absent from other cessation aids, such as being a part of a supportive vaping community. Empowerment and social support are closely associated with factors playing critical roles in smoking cessation such as improvement in mental health, quality of life, self-efficacy and self-esteem [44,45]. 

Many users also positively contrasted e-cigarettes with officially sanctioned quit smoking aids (nicotine replacement therapy and prescription medications), about which they vocalised their dislike [46]. Here again, participants’ discourses are corroborated by research demonstrating that e-cigarettes may be more acceptable than nicotine replacement therapy products owing to their sensory-behavioural similarities to cigarettes and superior efficacy at relieving cravings [16,47,48,49,50]. Although smoking cessation guidelines recommend that smokers with mental illness be closely monitored for neuropsychiatric adverse events when using varenicline and bupropion, evidence suggests that varenicline and bupropion are effective and tolerable for smoking cessation in people with mental illness [8,46]. Presently, there are no studies which compare the efficacy and adverse effects of e-cigarettes and varenicline or bupropion. It remains to be seen if the risk of neuropsychiatric adverse events is less for e-cigarettes, as these Reddit posters suggest. Our results also showed that some posters liked exploring different e-liquid flavors—a factor that may be an important part of the vaping experience that increases its desirability as an alternative to smoking [51]. This aspect of vaping contrasts with the more limited flavours available for nicotine replacement therapy, which smokers describe as being unpleasant [52,53]. 

Ease of use, affordability and safety were important factors brought up in deliberations on Reddit by both people with mental illness and their caregivers. E-cigarettes are a more complicated nicotine delivery system compared to a cigarette or nicotine replacement therapy and their use and maintenance is associated with a “learning curve” [54]. This may pose a barrier to use among those who experience lack of motivation (e.g., people with schizophrenia) or those who do not have supportive caregivers to help navigate the “learning curve” [43]. Although the variety of e-cigarettes available provides more choice and individualisation, it may be challenging (and costly) for people with mental illness to navigate the large number of options and configurations available. The initial outlay for a quality e-cigarette kit may make it economically unviable for smokers with mental illness who may be financially disadvantaged and unable to bear the costs of trialling different e-cigarette setups [7]. 

Posters raised concerns about the safety of psychiatrically unstable individuals possessing nicotine e-liquids who may accidentally or intentionally use it for self-harm [55]. While it is possible that refillable tank devices may be less suitable for people with cognitive deficits or self-harming tendencies, the simpler closed cartridge type of devices (first generation “cigalikes”) are generally less efficient in nicotine delivery than refillable tank devices and may not be a satisfactory replacement for smokers that have heavy nicotine dependence, as many people with mental illness have [55,56,57,58,59]. Research on which closed cartridge systems have the best nicotine delivery could assist smokers with mental illness to find the most suitable device.

### 4.1. Unofficial Sources of E-Cigarette Information

This study highlights the importance of Reddit as a site for the production and consumption of “lay expertise”—subjective experiential knowledge—about e-cigarettes for people with mental illness and their caregivers. Similar to our findings, previous studies have reported that non-clinical social interactions, both face-to-face and via social media constitute a significant source of information about e-cigarettes among the general population [22,60,61]. This kind of self-help approach for vaping finds its parallels in illicit drug harm reduction where expertise based on lived experiences and peer-to-peer interactions has contributed significantly to supplementing the specialist knowledge base and influencing the practices of those affected by substance use disorders [62,63]. Posts from health practitioners, carers and family members suggested that these posters wanted to encourage smokers with mental illness to switch to vaping. The physical and emotional proximity of health practitioners and other caregivers to smokers with mental illness, may increase their awareness of the difficulties this population group experiences with quitting smoking, providing valuable insights for researchers that may lead to exploration of new harm reduction strategies [64]. Support from family members and advice from health practitioners may predict smoking cessation and also influence the choice of smoking cessation aids [65,66,67]. The first line of treatment for smoking cessation in people with mental illness is provision of approved pharmacotherapy and intensive behavioural counselling [46]. However, for smokers with mental illness who have tried and failed to quit with these approved therapies, e-cigarettes may be the next recourse for reducing their smoking-related harm [68]. Some health practitioners may prefer not to recommend e-cigarettes to their patients due to the lack of evidence on their long-term effects and because they are not approved as cessation medicines. However, others might want to support their patients to use e-cigarettes to quit smoking, in view of the existing research which suggests that e-cigarettes can be used as a smoking cessation or harm reduction aid for smokers with mental illness [15,16,17]. Advice from health practitioners and caregivers who recommend e-cigarettes could be a significant motivation to take up vaping. However, being unable to obtain practical information about how to use e-cigarettes effectively for quitting smoking (e.g., most appropriate device, optimal nicotine strength, how to maintain the device etc.) from traditional sources (e.g., medical professionals, clinical guidelines) may encourage the use of online sources and lay expertise for this information.

### 4.2. Practice and Policy Implications

Bearing in mind the interest of people with mental illness and their caregivers in using e-cigarettes, health practitioners may be expected to provide detailed advice about e-cigarette use for quitting smoking [10]. The fact that multiple stakeholders, including mental health professionals were actively seeking information on Reddit may reflect the scarcity of practical information and training resources for health practitioners about how to use e-cigarettes to quit smoking [10,69]. In the absence of advice from healthcare professionals about e-cigarettes, prospective vapers may also rely more heavily on online sources such as Reddit and YouTube for this information [29,70]. Although some of this information may be accurate, the differing viewpoints presented (and its blending of evidential and subjective experiential knowledge), and targeted marketing of products via social media, may make it difficult for a non-expert to navigate the credibility of information in order to make an informed choice. Developing information resources about e-cigarettes for health practitioners as well as patients with mental illness that are trusted and easily accessible is an important challenge for health promotion. Analysis of online forums like Reddit can help inform the content of such resources by identifying the topics that are of importance to information seekers. For example, resources should include information about drug interactions, e-liquid safety, different configurations of e-cigarettes and which are most appropriate for people living with mental illness. The extensive interest in vaping calls for a possible trade-off between waiting for “certainty” from long-term studies versus the implications of not making practical advice available via official channels, if it leads health professionals and others to rely on information via peer-to-peer platforms like Reddit, which contains a mixture of both accurate and inaccurate information. 

### 4.3. Strengths and Limitations

We only collected data from one social media site. However, Reddit is one of the largest open online discussion boards. The approach was naturalistic insofar as we were able to analyze spontaneously and freely expressed views and needs, albeit at the cost of not being able to record demographic information for the participants or to ask questions to obtain greater clarity. Despite conducting a thorough search, we may have missed some relevant posts which did not appear in our searches. Nonetheless, we were able to capture a wide range of views about motivations and limitations of e-cigarette use in people with mental illness. Laws governing e-cigarettes vary between countries and sometimes between different jurisdictions within the same country. While it would be interesting to explore geographical differences in attitudes towards e-cigarettes, it was not possible with these Reddit data which do not identify the nationalities of posters.

## 5. Conclusions

This study shows that Reddit is widely used to deliberate upon and share information about issues surrounding e-cigarettes and mental illness. Posters discussed both positive and negative aspects of vaping. Our findings may be useful for policy makers, researchers, health communicators and health practitioners who may want to consider the pros and cons of vaping by people with mental illness when developing policy, guidelines and health promotion initiatives.

## Figures and Tables

**Table 1:** Characteristics of posters contributing to Reddit posts included in the analysis.

Characteristic	*n*
**Total number of individual posters**	1681
**Total number of individual comments**	3263
**Total self-identifying as having a mental illness**	413
**Carers**	42
	***n* (% out of 413)**
**Schizophrenia**	9 (2.1)
**Depression**	118 (28.6)
**Bipolar Disorder**	19 (4.6)
**Anxiety**	144 (34.9)
**Multiple diagnoses**	90 (21.8)
**Other**	33 (7.9)
	***n* (% out of 1681)**
**Vaper**	880 (52.3)
**Smoker**	41 (2.4)
**Non smoker**	60 (3.6)
**Former smoker**	444 (26.4)

**Table 2:** Motivations for using e-cigarettes.

Theme	Representative Quotes
***Self-medication***	“I have PTSD, anxiety symptoms from that, and TBI-related memory issues and micro seizures. For me, vaping is pretty much the same as smoking, in terms of how it helps me calm down and handle stress.”
	**(PI 1171)/r/electronic_cigarette**
	“My personal story? I didn’t smoke … cigarettes. I have problems with my mind, somewhere along the lines of depression and PTSD, and am allergic to the medications used to treat it … I had read previously reports that nicotine mildly helped people with conditions like mine. So I got an ego, a protank, and some juice and gave it a shot. Miraculously enough … I felt better.”
	**(PI 558)/r/electronic_cigarette**
	“Vaping has done wonders for my anxiety and depression; I was on 2 or 3 meds a day to treat them both. Keyword was.”
	**(PI 566)/r/electronic_cigarette**
	“I vape, no side effects except better concentration and appetite reduction—which counteracts the lithium.”
	**(PI 941)/r/bipolar**
***Quitting smoking***	“Just try to avoid the cancer sticks because I was more depressed on them than I am vaping. I love trying new flavours when I’m vaping and helps keep me interested. I usually vape 6 mg but I keep a few bottles of higher nicotine in case I am really feeling the need for nicotine. Best of luck to you!”
	**(PI 190)/r/electronic_cigarette**
	“Regarding the Chantix: I tried it, and have a history of depression. Had to stop it because I was bursting into tears at a minimum of 3 times a day. 5 or 6 was not unusual. The vivid dreams were freaking me out a bit as well: I often couldn’t determine what had actually occurred in real life, and what I had dreamt. So yeah, I’m with your doc. I’m using an Ego Twist with a Kanger Evod clearomizer, and have no issues…”
	**(PI 580)/r/electronic_cigarette**
	“As a Bipolar person it may be harder to quit smoking but it is very much worthwhile! I quit earlier this year and I feel so much healthier and naturally energetic now. Try to keep in mind how you will feel if you succeed: less worried about your health, breathe easier, able to exercise longer, food will taste better.”
	**(PI 384)/r/bipolar**
***Freedom and control***	“Now this kit was pretty good, I barely felt the nicotine, but I started to feel confident, and felt a lot of my anxiety drift away. I’ve been starting to regain control of my life; hell I’m even posting on reddit. Vaping not only saved my life, but freed me from a cage.”
	**(PI 906)/r/electronic_cigarette**
	“I have also battled with depression and anxiety. Fourteen years ago I picked up my first pack of cigarettes because it helped calm me down…I found Vaping about two years ago and gave it a try. I have been smoke free ever since and love the added bonus of being able to control my nic levels, low nic (3 mg) for my all day vape and high nic (9 mg) when I feel I need it.”
	**(PI 1042)/r/electronic_cigarette**
***Hobby***	“Vaping has helped improve my mental health but it wasn’t because of nicotine, it was because it gave me a simple hobby to focus on … Zen and the Art of Coil Building.”
	**(PI 400)/r/electronic_cigarette**
	“I vape 0 mg and I find it still helps with anxiety, depression, and concentration. It also gets me to stop snacking on so much bullshit when I get anxious or something. It really depends on you as a person. Some of us orally fixate or have nervous habits that vaping replaces.”
	**(PI 23)/r/Vaping101**
***Social connectedness***	“OK, here’s from someone who also suffers social anxiety, vaping has helped in more ways than quitting smoking. It’s a conversation starter. People will approach you. People will want to know what you’re doing. At first it’s overwhelming but over time it’s helped build my confidence in extreme ways.”
	**(PI 1517)/r/electronic_cigarette**
	“I was in the same boat as you OP, except cigs were a means to socialization for me when I was in school. By the time I turned 20 I was smoking a pack a day. Now I’m more than two months cig free, feeling so much better, and I have a new hobby/this generally awesome community for support!”
	**(PI 61)/r/electronic_cigarette**
***Motivation by caregivers***	“I never knew how easy it would be to switch from analogs to E-cigs. If it wasn’t for my buddy (friend’s name) I probably wouldn’t ever know. He had been vaping for a few months at this point. He had recently purchased a new iTaste MVP. I was talking to him about how broke I was and how expensive cigarettes were. I said I wish I could quit. He said I think I have something for you. Within 15 min He drove over a brand new EVOD kit and showed me how to use it.”
	**(PI 1246)/r/electronic_cigarette**
	“Have a renewed sense of self-worth and no longer feel like a second class citizen because I have a nicotine addiction that makes me a social pariah because of the smell and stigma attached to analogue cigarettes. Thank you so much to the/r/electronic_cigarette community for acting as a catalyst to such a positive change in my life!!!”
	**(PI 1529)/r/electronic_cigarette**
	“My Dads actually Bi-polar and smokes frequently. I really want to get him a good set up and see if he would like Vaping instead. Maybe if he switched it’ll keep his ass around a little longer lol.”
	**(PI 311)/r/electronic_cigarette**
	“Smoking at mental health facilities is illegal in Oregon yet many of the clients would still like to have something to “relax” them … My boyfriend has proposed ecigs at staff meetings and the company is looking into it but they work insanely slow…”
	**(PI 764)/r/electronic_cigarette**

PTSD, TBI are commonly used abbreviations for Post-Traumatic Stress Disorder, Traumatic Brain Injury.

**Table 3:** Limitations or barriers to using e-cigarettes.

Theme	Representative Quotes
***Unsatisfactory substitute for cigarettes and psychiatric medications***	“Vaping doesn’t really do it for me. That’s due to there being chemicals in burnt tobacco that function very similar to antidepressants (which is one of the big things that makes tobacco addicting). vaping doesn’t have those, and thus only has the effects of nicotine, which aren’t as strong.”
	**(PI 336)/r/electronic_cigarette**
	“I’ve had panic disorder since the age of 14. Celexa (and Xanax the first two weeks until it kicked in) helped me. I had a major relapse earlier this year and vaping certainly didn’t help. Had to go back to seeing a therapist and get back on Xanax until my increase in Celexa helped.”
	**(PI 851)/r/electronic_cigarette**
***Drug interactions***	“Hi all, I recently took up vaping (I enjoy it and it calms me—and helped me kick my weed habit) but I’m concerned that it may mess with my bipolar meds (1000 mg Depakote, 300 mg Seroquel, 300 mg Wellbutrin) if anyone has any experience with this and wouldn’t mind sharing their story I would be very grateful. Peace.”
	**(PI 1206)/r/bipolar**
	“So, I’m a pharmacist, and I would be glad to delve into your question … The problem? Well, if you are already taking medications that have similar effects, compounding the mechanism of actions (how your medication is working) will lead to increased risk of adverse effects. One of the most dangerous adverse effects from antidepressants is increased suicidal ideation. Other dangerous effects include serotonin syndrome, which often requires immediate hospitalization. This is the reason for the safety warning.”
	**(PI 257)/r/electronic_cigarette**
***Nicotine addiction***	“I have depression myself—if it helps you that’s awesome. My only real concern would be nicotine possible addiction even if it is in a far less harmful form. Aside from that though, I’m genuinely glad it’s helping you, regardless of nic content.”
	**(PI 1656)/r/electronic_cigarette**
	“Vaping MAY be healthier but nicotine is still nasty stuff on its own. It is especially bad as it hardens arterial walls, which has been linked to cardiovascular disease and death.”
	**(PI 663)/r/bipolar**
***Risks of e-liquids***	“I think what the OP means is that nicotine on its own is more poisonous than cyanide and arsenic. 60 mg will kill a light smoker, and I believe 45 mg is enough to kill many people who don’t smoke. Giving nicotine juice to someone with major depressive disorder may not be the best idea in the world.”
	**(PI 60)/r/electronic_cigarette**
	“I also think pre-filled would be the way to go. Clients getting nic all over their hands or mouth presents some serious trouble. Especially if they have certain issues, they could use it in some pretty negative ways (drinking it, etc.)”
	**(PI 764)/r/electronic_cigarette**
***Practical difficulties***	“My mother has schizophrenia … She has a terrible smokers cough and I think if I could get her to swap to e-cigarettes it would make a hell of a difference … Trouble is it needs to be dead simple. Even the recharging could cause problems and the refilling almost certainly would have to be done periodically by members of the family … Good charge and easy to charge. Maybe affordable enough to have a few so she can wait for a family member to refill or very easy to refill.”
	**(PI 1475)/r/electronic_cigarette**
	“I get juice on my hands and in my mouth frequently because I am an idiot. When I first started vaping, I accidentally screwed the bottom of my clearomizer off my ego twist and got 2 mL of 18 mg juice all over my hands. I just wiped it up, went to the kitchen and washed my hands in warm water with some dish washing liquid. Totally fine.”
	**(PI 70)/r/electronic_cigarette**
***Cost***	“I work in the mental health field, and smoking is a huge issue in terms of both the individual’s health and the safety of their environment. I’m looking for opinions on the easiest to use ecigs. Some individuals have tried Blu or Logic brands (convenience store ecigs) but they haven’t worked well in part due to the high cost ($10–$15).”
	**(PI 13)/r/electronic_cigarette**
	“Let’s not talk about money. I’ve fallen deep into the rabbit hole. Turn away and save yourselves, but it’s too late for me. Cigarette money is now going on vape gear. Anyway…”
	**(PI 1111)/r/electronic_cigarette**

OP is commonly used abbreviations for Original Poster.

## Supplementary Materials

The following are available online at www.mdpi.com/1660-4601/14/1/7/s1, Table S1: List of subreddits for the included comments. Table S2: Poster characteristics. Table S3: Motivations for using e-cigarettes. Table S4: Limitations or barriers to using e-cigarettes.
