# Supplementary material for: Motivations and Limitations Associated with Vaping among People with Mental Illness: A Qualitative Analysis of Reddit Discussions
Source: Int J Environ Res Public Health. 2016 Dec 22;14(1):7. doi: 10.3390/ijerph14010007 (PMC5295258; doi:10.3390/ijerph14010007)
Supplement: Supplementary file 1 [file ijerph-14-00007-s001.pdf]

Supplementary Materials: Motivations and Limitations Associated with Vaping among People with Mental Illness: A Qualitative Analysis of Reddit Discussions

Ratika Sharma, Britta Wigginton, Carla Meurk, Pauline Ford and Coral E. Gartner

Table S1. List of subreddits for the included comments.

Subreddits	Number of Comments—*n* (%)
/r/ADHD	15 (0.4)
/r/Anxiety	55 (1.6)
/r/aussievapers	35 (1.1)
/r/bipolar	39 (1.2)
/r/BipolarReddit	24 (0.7)
/r/Canadian_ecigarette	12 (0.4)
r/casualiama	7 (0.2)
/r/DecidingToBeBetter	14 (0.4)
/r/depression	43 (1.3)
/r/electronic_cigarette	2670 (81.8)
/r/Fitness	4 (0.1)
/r/Hidradenitis	7 (0.2)
/r/MMJ	4 (0.1)
/r/Narcolepsy	11 (0.3)
/r/NoFap	15 (0.5)
/r/RandomActsOfPizza	2 (0.1)
/r/running	68 (2.1)
/r/stopsmoking	116 (3.6)
/r/Vaping	29 (0.9)
/r/Vaping101	61 (1.9)
/r/vaporents	32 (1.0)

Table S2. Poster characteristics.

Diagnosis	Smoking Status	Vaping Status			
		Vaper	Non Vaper	Not Specified	Total
**Schizophrenia**	Smoker	0	0	1	1
	Non smoker	0	0	0	0
	Former smoker	6	0	0	6
	Not specified	2	0	0	2
Total		8	0	1	9
**Depression**	Smoker	0	1	0	1
	Non smoker	6	1	1	8
	Former smoker	48	1	8	57
	Not specified	31	3	18	52
Total		85	6	27	118
**Anxiety**	Smoker	3	0	4	7
	Non smoker	4	0	0	4
	Former smoker	52	0	3	55
	Not specified	44	3	31	78
Total		103	3	38	144
**Bipolar disorder**	Smoker	0	2	3	5
	Non smoker	0	0	0	0
	Former smoker	2	0	3	5
	Not specified	9	0	0	9
Total		11	2	6	19
**Other**	Smoker	3	0	0	3
	Non smoker	1	1	0	2
	Former smoker	11	1	2	14
	Not specified	10	0	4	14
Total		25	2	6	33
**Multiple diagnoses**	Smoker	1	3	0	3
	Non smoker	14	2	0	16
	Former smoker	35	3	4	42
	Not specified	21	0	8	29
Total		70	8	12	90
**Carers**	Smoker	1	2	1	4
	Non smoker	1	2	0	3
	Former smoker	9	2	1	12
	Not specified	7	0	16	23
Total		18	6	18	42
**Not specified**	Smoker	9	1	6	16
	Non smoker	23	2	2	27
	Former smoker	197	7	48	252
	Not specified	331	3	597	931
Total		560	13	653	1226
**Total**	Smoker	17	9	15	41
	Non smoker	49	8	3	60
	Former smoker	360	14	69	443
	Not specified	454	9	674	1137
Total		880	40	761	1681

Table S3. Motivations for using e-cigarettes.

Subtheme	Representative Quotes
Self-medication	"I had tried smoking since people have said that nicotine can act as an antidepressant that isn't really that bad for you. I did it a couple of times yet was disgusted with the destruction it had on my body. Then a few months ago I heard of vaping—I picked up my first ego style vape pen with a low nic level and enjoyed it. Eventually, I upgraded to a subox mini and vape on 3 mg." **(PI 927)/r/electronic_cigarette**
	"I've been smoking for the better part of a year or so. That is, until around a week ago, when I picked up my MVP 2.0. I have an anxiety disorder and moderate depression, and have frequent suicidal thought … On payday I bought my MVP, and I have never looked back. All the suicidal ideations have vanished (thanks nicotine) and I'm more focused and relaxed than ever! I can't thank you guys enough for showing me the light." **(PI 153)/r/electronic_cigarette**
	"I'm a non-smoker that bought an E-Cig to combat anxiety and depression. It works: So I've never been a smoker, but I learned that low doses of nicotine can help with anxiety and depression. I was worried that I would become addicted to nicotine or that it just wouldn't work. Well I'm happy to say that during and after each vaping session, I noticed that negative thoughts just seemed to vanish." **(PI 331)/r/Anxiety**
	"I have really bad anxiety, but don't really like the idea of going on medications too (sic) combat it since it messes with your brain's natural chemistry in both bad and good ways. Vaping and being able to choose/vary what levels of nicotine I wish to use to have a sort of desired "calming" effect has really helped out greatly with my issues. Also vaping has really effected my social anxiety issues as well." **(PI 766)/r/electronic_cigarette**
Quitting smoking	"Depression and Anxiety- Two weeks semi analogue free. I already feel better, one of my most notice things is that I have an easier time getting out of bed in the mornings. I also vape way less than I smoked…I've noticed a huge increase in stamina and breathing—Mo' money, so easier to purchase my medication." **(PI 412)/r/electronic_cigarette**
	"I quit through vaping, not just a little tiny one but it's good to invest on something expensive. Although you're trading one addiction for another, it's the lesser of the two evils. I was a pack a day smoker and I quit within two weeks." **(PI 443)/r/bipolar**
	"The mods and juices are so cheap that it is like it is non-existent to my budget. I don't have to skip on dates like when I was taking concerta. I don't get cranky or smell bad like when I was smoking. This pure form of nicotine is a force to be reckoned." **(PI 1039)/r/electronic_cigarette**
	"3 years of e cigs, vape pens and different devices I haven't had a cigarette in months. My nicotine dependency is next to gone and I feel great. It opened a door for a new hobby during a really bad depression. I had something to be enthusiastic about and interested in." **(PI 237)/r/electronic_cigarette**
	"Hello! I am a former smoker now vaper with severe agoraphobia and anxiety disorders. I've used smoking as an anti-anxiety tool for a decade, since I started vaping (18 mg juice for my coffee drinking sessions and 12 mg juice for chain vaping) I have not had the horrible panic I had when I tried to quit with gum and lozenges. I find vaping keeps me a little more even and levelled out through the day, and in times of stress, hiding in a corner with some 18 mg juice for a while has proven a just as effective as chain smoking half a pack of cigarettes." **(PI 70)/r/electronic_cigarette**

Table S3. *Cont.*

Subtheme	Representative Quotes
**Quitting smoking**	"I had tried Zyban many years ago. I practically flipped personalities on it. I'm typically extremely patient and calm but on that shit I was beyond quick tempered and easy to aggravate. I wouldn't go near Champix." **(PI 92)/r/Canadian_ecigarette**
	"As an aside, my mother tried quitting smoking with patches, gum, etc. etc. and those REALLY fucked with her medications and/or depression. So if you're using your vaporizer to quit smoking, I imagine it would work much better and wish you the best of luck." **(PI 521)/r/electronic_cigarette**
	"I find vaporizing to be best. Smoking has a lot of health risks, the patch/gum/snus did nothing for me." **(PI 1182)/r/electronic_cigarette**
	"I'm a frequent flier at "Happy Camp." That's how my family and I jokingly refer to the mental health floor at the hospital. Regular as clockwork, I think life is out of control every five years or so. The last time, 2009, we were still allowed to go outside (up on the roof LOL) twice a day to smoke. I'm guessing that won't be the case next year when I'm ready for an emotional oil change. I've definitely got to get to vaping full time by then. I don't know that they will allow vaping, but I figure it is a much better bet than smoking." **(PI 1502)/r/electronic_cigarette**
**Freedom and control**	"I've been vaping for a little over 2 months. About a month ago, I started to taper off my anti-anxiety meds. Partly because I have been taking them for so long, partly because I just don't like taking pills. Anyway, I am off the meds entirely now and I have not had any sort of relapse into an anxiety attack. All my old triggers that I have faced have not had an effect. I am pleased to have the independent feeling and being in control again. I can't say vaping has helped, maybe it has, I have no idea. But it sure didn't hurt anything as far as I can tell. For the record, I started at 12 mg nic for about a week, then switched to 6 mg for the remainder of that time." **(PI 184)/r/electronic_cigarette**
	"Thank you for sharing your story. I have also battled with depression and anxiety. Fourteen years ago I picked up my first pack of cigarettes because it helped calm me down. I always hated how cigarettes made me feel however after smoking for eleven years I thought that I was hooked for the rest of my shortened life however I found Vaping about two years ago and gave it a try. I have been smoke free ever since and love the added bonus of being able to control my nic levels, low nic (3 mg) for my all day vape and high nic (9 mg) when i feel I need it. Living with even mild depression and/or anxiety sucks I'm glad that you have found something that helps you." **(PI 1042)/r/electronic_cigarette**
**Hobby**	"I've found that vaping as a hobby/leisure activity has really helped with my depression as well. Rebuilding can be very relaxing and having vape mail to look forward to is always a perk." **(PI 1618)/r/electronic_cigarette**
	"When I first started vaping, I didn't use any nicotine but after buying juice with nicotine, I've noticed my own anxiety as well as my depression not being as bad. It's something to do with my hands and keeps me busy.<br>To note, I've had chronic depression for about a year and a half now and vaping has certainly helped." **(PI 543)/r/electronic_cigarette**

Table S3. *Cont.*

Subtheme	Representative Quotes
**Hobby**	"Vaping works for my anxiety because I'm a fidgeter and a comfort eater. I need something to do with my hands, and often that something is to put things in my mouth. Vaping satisfies both of those comfort mechanisms. I can get the same effect with a Rubik's Cube and a lollipop, except I'm diabetic so the lollipop is a terrible idea." **(PI 955)/r/electronic_cigarette**
	"I'm really glad you found something that helps you and that you are feeling better! A hobby like vaping can really help you, I know it has me." **(PI 534)/r/electronic_cigarette**
**Social connectedness**	"I have pretty severe anxiety and vaping helps with that a TON for me. Whenever I start to vape maybe it's the act of vaping itself or the combination of low-levels of nicotine (3 mg) but it really, really calms me down. Either way I've been a lot happier since I've been vaping! It helps me be more social too and meet new people with such an awesome common interest. Thanks for sharing your story." **(PI 186)/r/electronic_cigarette**
	"I appreciate this and for sharing your experience—I am so overwhelmed by the positivity from this community and have truly found that I am not alone in this. Thank you." **(PI 927)/r/electronic_cigarette**
	"I've always had a problem with social anxiety and meeting new people, and vaping has helped me with that. I now have something to relate and to talk about with people." **(PI 534)/r/electronic_cigarette**
	"Also vaping has really effected my social anxiety issues as well. Since I began vaping (and vaping in semi-public areas) I've been approached by numerous happy people who just LOVE to talk about vape and such, as they're so happy to have found someone who shares their hobby. I've made a couple good friends through vaping, and have also found this amazing place! We all have our own reasons to vape it seems!" **(PI 766)/r/electronic_cigarette**
	"It helps with my anxiety and in general keeps me from wanting top killing myself! I'm not afraid to say it I have tried in the past but with the help of vaping i don't feel low. And I have met this community, ya y'all are a bunch of assholes but we are united by the fact that we all vape and want to help one another so thank you every one. I look forward to another year with you guys (and gals)." **(PI 1009)/r/electronic_cigarette**
**Motivation by caregivers and online communities**	"I have never touched a cigarette in my life. I tried dipping once and hookah a few times and got sick every time. However I suffer from depression and anxiety and my hands shake uncontrollably at times. One day a friend of mine who is a former smoker who turned to vaping had me take a few hits from his vape, and the shakes in my hands subsided. I eventually got my own mod and subscribed to zamplebox, and I tell you, it has helped big time with the shakes in my hands. I never go above level 3 nicotine either. I just wanted to share my story and this seemed a good place to do so. Thanks for reading." **(PI 1301)/r/Vaping101**
	"I know for me, quitting a 15 year long cig addiction sure helped my depression. And having discovered a really fun hobby with an amazingly engaging and supportive community has been the best part of vaping." **(PI 1176)/r/electronic_cigarette**
	"Immediately, most of the clinicians said that they're proud of their clients for kicking the harmful analogue, and some even noted how they enjoy the way their offices smell like cinnamon and cupcakes instead of ash trays." **(PI 306)/r/electronic_cigarette**
	"I am an active duty military psych-tech. Having worked at an inpatient facility I wish that the government would allow vaping as an alternative." **(PI 1503)/r/electronic_cigarette**

Table S4. Limitations or barriers to using e-cigarettes.

Subtheme	Representative Quotes
Unsatisfactory substitute for cigarettes and psychiatric medications	"I've tried getting my mentally ill brother interested in e-cigs but haven't had any luck. He thinks they're a joke. Even if was interested, I don't know if he could handle the refilling, cleaning and tweaking required for most e-cigs. He has no patience and no tolerance so even with a pre-filled system, I could see him easily tossing 5 or more prefilled cartos in the trash each day." **(PI 675)/r/electronic_cigarette**
	"A therapist would be a much better idea. I have anxiety also and if anything, vaping makes it worse. At least for me." **(PI 21)/r/electronic_cigarette**
	"Breaking a cigarette habit in this case may be more difficult because of the lack of MAOIs in e-cigs. It's hypothesized that schizophrenics self-medicate with tobacco due to the MAOIs that are found in the smoke. (Hence high rates of smoking in these patient populations)." **(PI 1377)/r/electronic_cigarette**
Drug interactions	"I recently upgraded my vaporizer and got new e-juice. On the e-juice packaging it says I shouldn't use it if I'm taking depression medication. Since I'm currently on antidepressants, I've been searching for information on contraindications between the two. Have you guys heard anything along these lines?" **(PI 1388)/r/electronic_cigarette**
	"Ask your psychiatrist, but in my narrow experience I'm not aware of any issues. I've used nicotine for the same reasons on 1500–1800 mg lithium, 200 mg Lamictal, 400–500 mg Seroquel, 300 mg Wellbutrin, and 80 mg Strattera. I'm glad we have the vaping option." **(PI 1463)/r/bipolar**
Nicotine addiction	"I'm sorry if this sounds harsh but I like to keep things short. A nicotine addiction is not something you should pick up. Once you have one then you will be worse off than you are now." **(PI 951)/r/electronic_cigarette**
	"…I feel that the e-cig will be nothing more than a crutch, enabling me further down the chemical dependency spiral." **(PI 1056)/r/stopsmoking**
Risks of e-liquid	"I sometimes tend to over vape when upset (like chain 10+ hits at a time) 6–9 mg juice and it nearly sedates me physically and emotionally to the point of not being able to sit without having to vomit. This stuff will help, and it won't be as bad as anti-depressants, but it also won't work as well as they do (through the potential to have serious addiction that can lead to overdose is massive with pharma anti-depressants)." (**PI 1223)/r/electronic_cigarette**
	"I used to own one of those electronic cigarettes but I actually found they gave me severe chest pains (I know right? and cigarettes do not.) I really wish I had one that didn't give me chest pains." **(PI 1352)/r/stopsmoking**
	"I just changed from a 1.0 ohm to a 0.5 ohm and with the same e-juice and now the nicotine is burning my throat/mouth a bit. Hopefully I get used to it. As habits go, I feel vaping is probably one of the least detrimental, but you should be aware that, of course, with the lack of research, none of us can be 100% certain of the long-term effects." **(PI 906)/r/electronic_cigarette**

Table S4. *Cont.*

Subtheme	Representative Quotes
Practical difficulties	"I wear gloves now when I make my juice, because the nic does affect my skin if I don't wash it off right away. It didn't kill me obviously, but it does create little callous looking areas where the juice has been." **(PI 220)/r/electronic_cigarette**
	"The majority of the folks in my program don't have the ability to use eGo's with anything requiring re-filling. A simple, non-VV/VW eGo or other type 510 with pre-filled carto's would probably be ok though. It's a matter of loss of dexterity (often due to the medications they take) and a lack of ability to focus on the refilling procedure. I'm speaking in general here, some folks could handle it, but probably not the majority." **(PI 13)/r/electronic_cigarette**
Cost	"You can vape if you want but that's pretty expensive, there's studies that it's just as bad, and it taste better so it'll be easier to like and do more often." **(PI 1446)/r/bipolar**
	"…but then again I do want to try out the 0 mg nicotine e cigs but currently I am broke(the disadvantages of being an aspiring music producer) and can't afford the expenses related to vaping. But I will try my best to relax and keep my calm. And thank you)." **(PI 1056)/r/stopsmoking**
	"As I highlighted above, these people are poor. They give up all of their spending money on the cheap little cigars and rolling machine cigarettes. Where would I ever begin to suggest how to go about getting them started on e-cigarettes?" **(PI 181)/r/electronic_cigarette**

© 2016 by the authors; licensee MDPI, Basel, Switzerland. This article is an open access article distributed under the terms and conditions of the Creative Commons by Attribution (CC-BY) license (http://creativecommons.org/licenses/by/4.0/).
